# Optimization and performance of nitrogen-doped carbon dots as a color conversion layer for white-LED applications

**DOI:** 10.3762/bjnano.10.197

**Published:** 2019-10-15

**Authors:** Tugrul Guner, Hurriyet Yuce, Didem Tascioglu, Eren Simsek, Umut Savaci, Aziz Genc, Servet Turan, Mustafa M Demir

**Affiliations:** 1 Department of Materials Science and Engineering, Izmir Institute of Technology, Izmir, Turkey; 2 Quantag Nanotechnologies, Istanbul, Turkey; 3 Department of Materials Science and Engineering, Eskisehir Technical University, Eskisehir, Turkey; 4 Department of Metallurgy and Materials Engineering, Bartın University, Bartın, Turkey

**Keywords:** color conversion layer, electrospinning, nitrogen-doped carbon dots, PVP, solid-state lighting, white LED, white-light generation

## Abstract

In this study, green-emitting nitrogen-doped carbon dots (N-CDots) were synthesized and incorporated into drop-cast composite films for use as color conversion layers in a white-LED configuration to generate white light. In order to resolve the red deficiency of this configuration, a commercial red phosphor was integrated into the system. Moreover, the N-CDots were also processed into polymer/N-CDot composite fibers, for which we determined the amount of N-CDots that yielded adequate white-light properties. Finally, we showed that white light with excellent properties could be generated by employing both of the fabricated N-CDot composites either as drop-cast films or composite fibers. Hence, N-CDots provide a promising alternative to inorganic phosphors that are commonly employed in white-LED configurations.

## Introduction

Light-emitting carbon dots (CDots) are a new class of nanomaterials showing unique optical performance characterized by a wide wavelength tunability, high quantum yield and high photostability, while they show low toxicity and can be produced easily and at low cost [1–5]. The combination of such significant features renders CDots attractive for various applications, including bio-imaging [6–7], drug and gene delivery [8], sensors [9–10], photocatalysis [11], energy storage [12–13] and white-light-emitting diodes (WLEDs) [14–15]. Typically, these materials contain an internal carbon core, conjugated sp^2^ domains and some functional groups attached to their surface. The emission corresponding to such a structure is considered to originate from the conjugated sp^2^-domains [16–17] and can be adjusted either by modulating the dimension of these sp^2^-domains [18] or through surface modifications [19–20]. For instance, doping of the CDots with heteroatoms such as nitrogen can lead to a wavelength adjustment of the corresponding emission [21–23]. However, the details of the mechanism behind the optical characteristics of these materials are still being theoretically and experimentally explored by researchers.

The use of WLEDs, as a well-established component of solid-state lighting, provides a promising strategy to reduce electricity consumption, and WLEDs are expected to replace traditional lighting products in the future [24–27]. A WLED mainly consists of a blue or UV LED chip and phosphors as color conversion layers [28–32]. A typical configuration is the combination of a color conversion layer composed of yellow phosphors (e.g., cerium-doped yttrium aluminum garnet (YAG:Ce^3+^)) with a blue LED chip [33–37]. However, this configuration exhibits a low color rendering index (CRI) due to its red deficiency. Therefore, red phosphors can be integrated into this system to improve the CRI and also to reduce the correlated color temperature (CCT) [28,38]. However, due to the limitation of rare-earth material resources and the harsh synthesis conditions involved, phosphors are targeted to be replaced by novel materials such as perovskites [39–41], organic dyes [42–43], quantum dots [44] and CDots [15,45]. Among these, CDots are a promising alternative since they are cost effective and optically feasible, making them commercially viable.

Employing CDots as color conversion layers in a WLED configuration is a relatively new concept, since the first discovery of these materials dates back only to 2004 [46]. Since then, CDots have been widely studied. For instance, Feng et al. reported on the synthesis of carbon quantum dots for use as single light converters in WLEDs [45]. CDots were prepared by a one-step hydrothermal method using glucose and polyethylene glycol 200. It is reported that the WLED showed a superior performance indicated by Commission Internationale de l'Éclairage (CIE) coordinates of (0.32, 0.37) and a CCT of 5584 K. In another study, Joseph et al. reported the synthesis of CDots by the electrochemical exfoliation of graphite [47]. The obtained CDots showed a broad band emission due to their heterogeneity in particle size and surface-functional groups. The authors explored the potential of CDots as UV-to-visible color converters using a 365 nm UV chip and could generate white light with CIE color coordinates of (0.35, 0.37), a CRI of 88 and a CCT of 4802 K. Furthermore, Xie et al. fabricated WLEDs using polymerizable silane‒prefunctionalized carbon dots (Si-CDots) [48]. These green low-cost polymerizable Si-CDots produced by a one-pot electrochemical synthesis have emission peaks located around 541–549 nm. These Si-CDots were used as the color conversion and encapsulation layers of a commercially available GaN blue LED chip. The authors report that the luminous efficacy of radiation (LER) of the resulting Si-CDot WLED is 70.93 lm/W, the CIE coordinates are (0.28, 0.35), and the CRI is 69. In another work, novel nitrogen-doped multiple-core@shell-structured AC-CDots (AC: Aphen and Citric acid) with a tricolor emission comprising red, green, and blue were, for the first time, synthesized via a one-pot hydrothermal approach by Zhang et al. [49]. The authors further demonstrated that by combining the blue (430 nm), green (500 nm) and red (630 nm) fluorescence, the AC-CDots can produce a pure white-light emission spectrum. Finally, highly efficient CDot/gel glass, phosphor-based, trichromatic WLEDs were developed by Yuan et al. [50]. The authors doped green and red CDots into a matrix of methyltriethoxysilane and 3-(triethoxysilyl)propylamine, respectively, to prepare the CDot/gel glass. With this strategy, the authors achieved a LER of 71.75 lm W^−1^ and a CRI of 93. Lastly, red emitting [51] and white emitting Cl-doped CDots have also been reported [52].

In this work, we report the synthesis of nitrogen-doped CDots (N-CDots). The doping with nitrogen enables us to tune the resulting emission color of the CDots from blue to green. In order to obtain color conversion layers with these N-CDots, a mixture of water-based polyvinylpyrrolidone (PVP) and N-CDots were prepared and subsequently processed into thin films and fibers. Throughout this study, the term “water-based” is used to emphasize that during the fabrication of the color conversion layers, the CDots and the PVP were taken from their dispersion or solution in (mainly) distilled water without introducing any toxic or organic solvents. The conversion layers were prepared in the form of drop-cast composite films with varying amounts of CDots in order to investigate the effect on the resulting white-light properties. Moreover, commercial inorganic red phosphors were integrated into the PVP/N-CDot composite films to explore the impact of the red emission component on the white-light properties. Additionally, composite fibers were prepared by electrospinning of the water-based PVP/N-CDots. By adjusting the amount of fibers in the color conversion layers, the quality of the generated white light was improved, and a high CRI together with a low CCT were achieved.

## Experimental

### Materials and methods

Diammonium hydrogen citrate and urea were purchased from Sigma-Aldrich (St. Louis, MO, USA) and used without further purification. PVP (*M*_W_: 58,000, Alfa Aeasar) was used in the form of the polymer matrix. Commercial red phosphor, Sr_2_Si_5_N_8_:Eu^2+^, was purchased from Zhuhai Hanbo (HB-640, Guangdong, China). High-resolution transmission electron microscopy (HRTEM; JEOL 2100F, operated at 200 kV) was employed to examine the morphology of the N-CDots. X-ray photoelectron spectroscopy (XPS) studies were performed using a Thermo Scientific K-Alpha XPS spectrometer. Fourier-transform infrared spectroscopy (FTIR; Spectrum 100, PerkinElmer, Shelton, CT, USA) was used to characterize the kinetic behavior of the chemical bonds. Photoluminescence (PL) and absorbance were measured using an integrating sphere (ISP-50-80-R, Ocean Optics Inc.) attached to a USB2000+ spectrometer (Ocean Optics Inc., Dunedin, FL, USA) with a premium ﬁber cable. A commercial blue LED chip (CREE, 450 nm, royal blue) was used as the blue light source. The morphology of the produced N-CDot fibers was analyzed by scanning electron microscopy (SEM; Quanta 250, FEI, Hillsboro, OR, USA). Lastly, CRI, CCT, luminous flux and LER were calculated using the OSRAM ColorCalculator program. The quantum yield was obtained by using a fluorometer (Horiba Scientific Fluorolog) with a quanta-φ integrating sphere. We use remote-phosphor configuration, which color conversion layer(s) placed a particular distance away from LED chip, throughout this study. Details of this configuration can be found in our previous study [35].

### Synthesis of nitrogen-doped carbon dots

**Figure 1:**
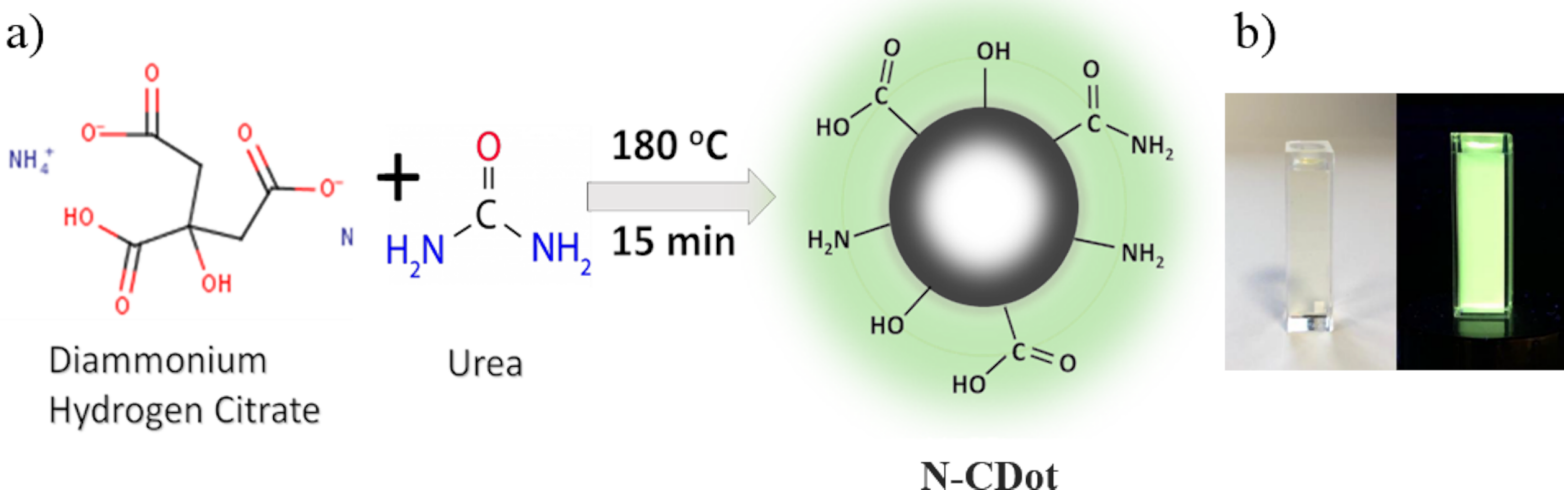
(a) Schematic illustration of the synthesis of the N-CDots and (b) photographic image of the diluted N-CDot suspension under natural light (left) and UV light (right) illumination.

Diammonium hydrogen citrate and urea were selected for the synthesis of nitrogen-doped CDots. First, diammonium hydrogen citrate and urea were mixed at a weight fraction of 1:1 and homogeneously ground. The mixture was subsequently heated under reflux for 15 min in air without any solvent. After the reaction, the mixture was purified by centrifugation at a rotation rate of 6000 rpm for 20 min [53]. Finally, the resultants were dispersed in distilled water (DW) such that a solution of CDots/DW was obtained at a concentration of 9% with a quantum yield of 16%. The yield of this synthesis procedure was found to be 55–60%. The overall process is summarized in Figure 1a. In this process, urea was chosen as the nitrogen source since it is cost-efficient, abundant and nitrogen-rich [54]. Photographic images of the resulting N-CDot suspension illuminated under both natural and UV light are presented in Figure 1b.

### Fabrication of nitrogen-doped carbon dot films and fibers

#### Production of the composite films

The PVP solution was prepared by dissolving 3.0 g of PVP in 10.0 mL of DW. Then, 1.0 mL of the PVP solution and the desired amount of the green N-CDot solution was homogenously mixed in a glass vial. The resulting mixture was drop-cast onto a glass slide to produce the thin composite films.

#### Production of the fibers

At first, 3.0 g of PVP was mixed with 2.5 mL of ethanol and 2.5 mL of pure water in a glass vial. Then, 2.0 mL of the PVP solution was mixed with 15.0 µL of the N-CDot dispersion for electrospinning. The PVP/N-CDot fibers were fabricated by employing a potential difference of 15 kV and a flow rate of 1 mL per hour as the operating conditions of the electrospinning process. Owing to the potential difference between the syringe tip and the collector aluminum foil, the N-CDots were confined into sub-micrometer fibers by electrospinning.

## Results and Discussion

### Structural characterization of the N-CDots

**Figure 2:**
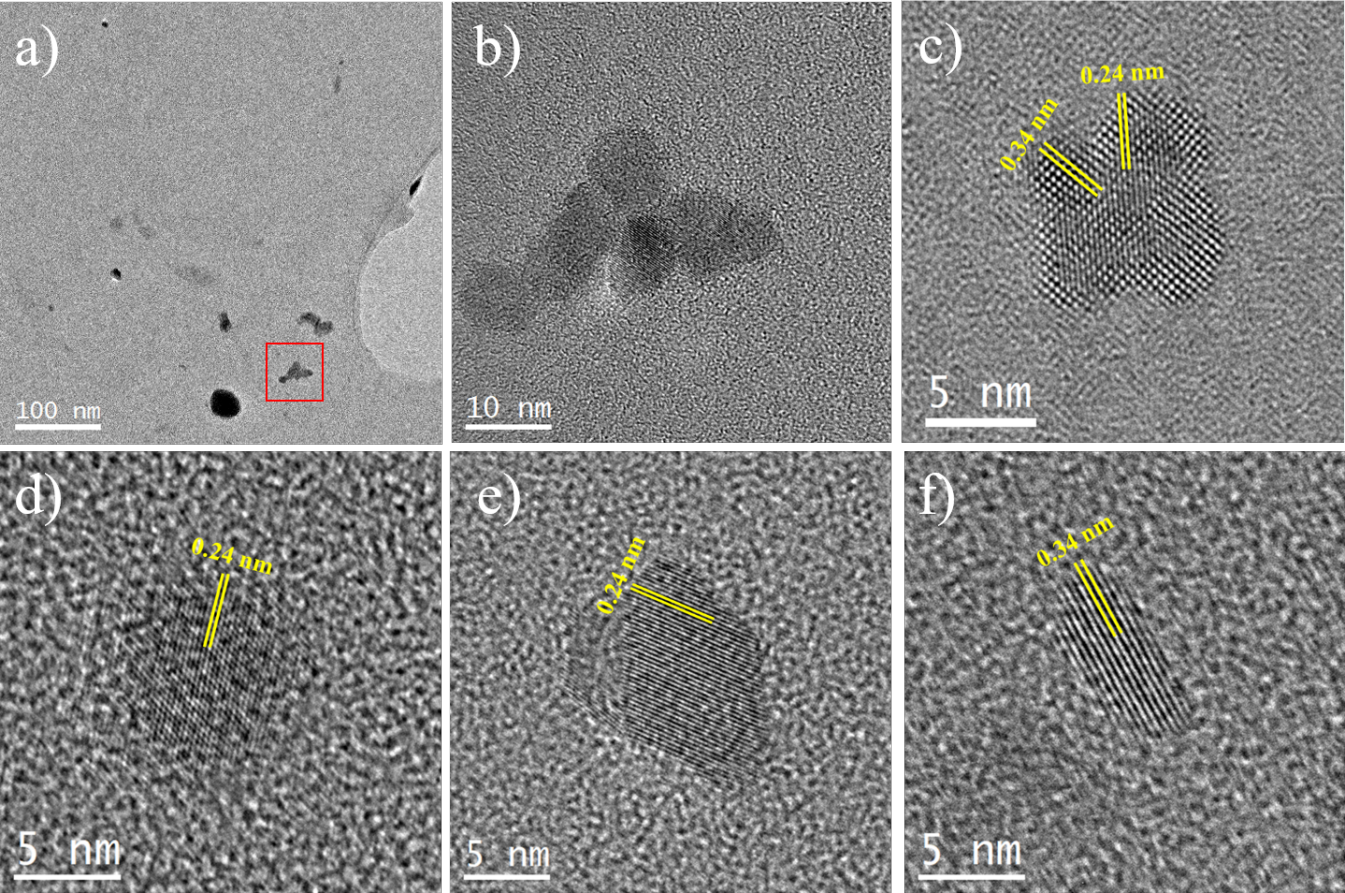
a) and b) General TEM micrographs of the green carbon quantum dot sample, showing the presence of small nanoparticles, and c–f) HRTEM micrographs of several individual nanoparticles identified as carbon quantum dots, where the 0.34 nm lattice spacing value corresponds to the (002) plane, and the 0.24 nm lattice spacing is formed by overlapping two (002) planes.

Figure 2 shows HRTEM micrographs of several individual nanoparticles identified as carbon quantum dots (CDots). We further diluted the stock solution for the HRTEM analysis in order to obtain the crystal structure of individual CDots avoiding possible agglomerations. As a consequence, the prepared TEM grid had a very low particle density and we could not obtain any statistical data on the particle size from the low-resolution TEM micrographs. The nanoparticles are mainly of between ≈4 nm and ≈10 nm in diameter, with an occasional presence of nanoparticles as large as 30 nm. The TEM micrograph presented in Figure 2b is the magnified image of the red squared region shown in Figure 2a. Figure 2b reveals that the cluster of about 40 nm in size, which is seen in Figure 2a, is formed by at least seven sub-10 nm nanocrystals. It is convenient to present *d*-spacing values for the identification of these nanoparticles instead of power spectra, which show diffraction spots generated by only one plane. Figure 2c shows the HRTEM micrograph of a ≈10 nm diameter nanoparticle. This nanoparticle is identified as being composed of a graphite phase based on the observation of the 0.34 nm lattice spacing values, corresponding to the (002) plane interlayer distance of the graphitic carbon phase (*P*6_3_*mc* space group) with lattice parameters of *a* = 0.246 nm and *c* = 0.671 nm. The lattice spacing value of 0.24 nm is obtained by overlapping two (002) planes as described in the literature [18]. Some other studies identified this lattice spacing as the {1120} lattice plane of graphene [55–56]. It should be noted here that when it comes to CDots, it is common to assign them as graphene rather than graphite, since typically, stacking of a few layers of graphene forms the CDots. The HRTEM micrographs obtained from a ≈9 nm nanoparticle (Figure 2d) and a ≈11 nm nanoparticle (Figure 2e) reveal the same lattice spacing of 0.24 nm for both nanoparticles. The HRTEM micrograph obtained from an elongated nanoparticle with a volume of 4 × 9 nm^2^ is shown in Figure 2f, revealing the presence of 0.34 nm lattice fringes corresponding to the (002) plane. Further information on the characterization of the CDots together with an extended discussion of the XPS results (Figure S1), the results of the FTIR study (Figure S2a) and absorption and PL spectra (Figure S2b) can be found in Supporting Information File 1 (Section I).

### White-light properties of the N-CDots in PVP

**Figure 3:**
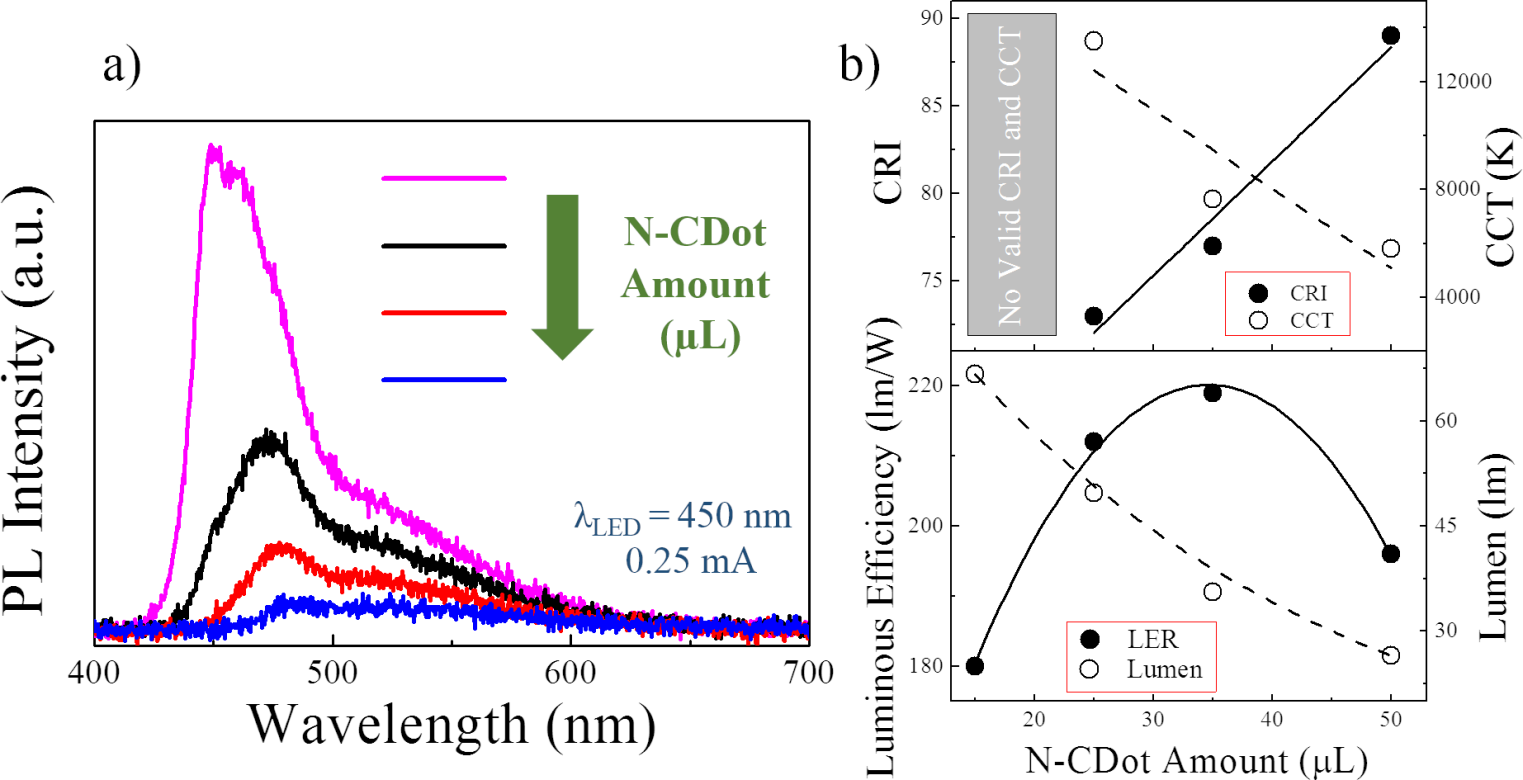
(a) PL spectra of white light generated by employing the PVP/N-CDot drop-cast films layered over a 450 nm blue LED chip as a function of the N-CDot amount (µL) (pink line: 15 µL, black line: 25 µL, red line: 35 µL and blue line: 50 µL). (b) Calculated CRI, CCT, LER and luminous flux (lumen) values of the related drop-cast films with different amounts of N-CDots.

The PVP/N-CDot composites were prepared and drop-cast on glass slides using a fixed PVP content but varying amounts of N-CDots ranging from 15 to 50 µL. To investigate the effect of the N-CDot concentration on the resulting white-light properties, these composites were employed as color conversion layers over a blue LED chip (driving current of 0.25 mA). Figure 3a presents the PL spectra of the drop-cast green PVP/N-CDot composites on the glass slides. There are two different signals: the first one, at 450 nm, originates from the blue LED source, and the other one, at around 506 nm, from the emission of the N-CDots. As the N-CDot concentration increases, both the PL intensities of the blue LED light and the N-CDots decrease. However, comparing their relative intensity, the ratio of the blue and green emission peaks changes upon increasing the amount of the N-CDots. At the lowest N-CDot amount of 15 µL (pink line), the blue emission is dramatically more intense than the green emission. Their ratio decreases as the amount of N-CDots increases since the reduction of the blue emission proceeds faster than the enhancement of the emission of the N-CDots, and this ratio becomes almost one at the highest amount of N-CDots (50 µL, blue line). Meanwhile, the PL intensity of the resulting PVP/N-CDot composites drops dramatically as the amount of the N-CDots increases. Such a reduction is expected due to aggregation-induced luminescence quenching (AILQ) [57–59], enhanced scattering, and the gradual transparency loss of the PVP, which increases the backscattering of light from the sample towards the LED. In Figure 3b, the white-light properties, such as CRI, CCT, and LER, that represent the efficacy of the produced light on the human eye (calculated by considering the human eye sensitivity [35,60]) together with the total visible light output (luminous flux) are presented as a function of the N-CDot amount. At the lowest N-CDot amount (15 µL), there is no valid registered CRI (filled circles) or CCT (empty circles) output. As the amount increases, the CRI increases linearly (solid line), while in contrast, the CCT follows a linearly decreasing pattern (dashed line). Therefore, the highest CRI of 89 and the lowest CCT of 5800 K have been obtained for the PVP composite containing the highest amount (50 µL) of green N-CDots. The LER value, presented as filled circles, follows a parabola function that reaches a maximum of 219 lm/W at a N-CDot amount of 35 µL. From 15 µL (180 lm/W) to 35 µL, the parabola has a positive slope, which is expected due to the decreasing blue-to-green ratio. However, at the highest amount of N-CDots, even though the blue-to-green emission ratio approaches unity while the overall emission reaches the lowest level, the resulting LER is reduced again to only 196 lm/W. The reason for this decrease may be the very low PL intensity (blue line in Figure 3a), which results in a low signal-to-noise ratio (*S*/*N*). The low *S*/*N* possibly causes an efficiency loss in the produced visible light output. Meanwhile, the luminous flux (empty circles) of the produced white light decays exponentially (dashed line) from 66.7 to 26.5 lm as the N-CDot amount increases. This behavior is expected since the total PL intensity of the PVP/N-CDot composites decreases dramatically as the N-CDot amount increases. Furthermore, the stability of the white-light properties of the prepared PVP/N-CDot composites against the driving current was also investigated (Supporting Information File 1, Figure S3 in Section II).

**Figure 4:**
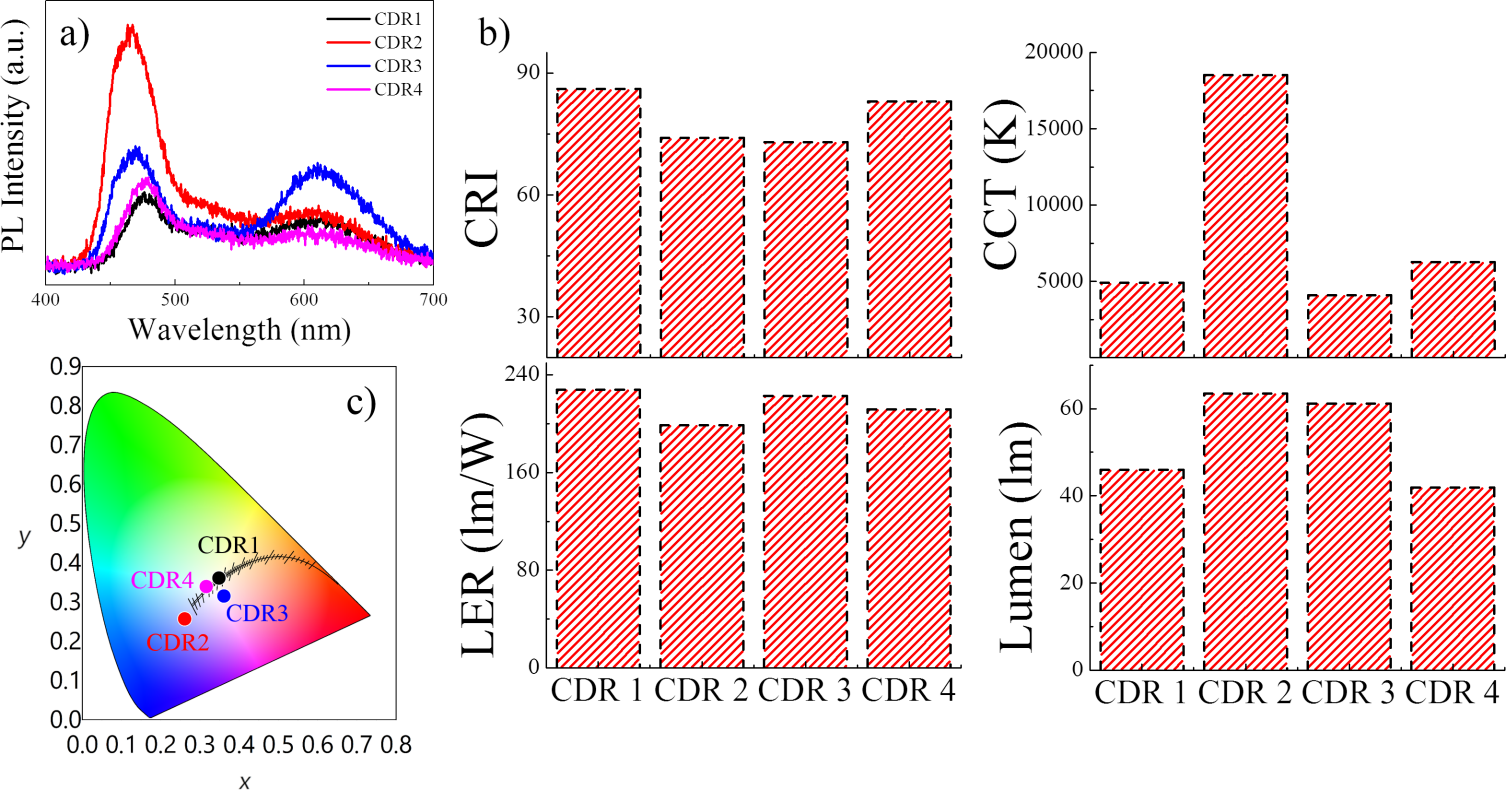
(a) The PL spectra of the WLED containing the drop-cast PVP/N-CDot films and inorganic red phosphor as color conversion layers over a 450 nm blue LED chip. The amounts of N-CDots and red phosphor within the composite films are varied. (b) The calculated CRI, CCT, LER and luminous flux of the color conversion layers. (c) The CIE color coordinates of the white light generated by the color conversion layers.

Using solely the PVP/N-CDot composites as color conversion layers, we have improved the CRI and the CCT values by adjusting the amount of N-CDots. The LER adopts a distinct maximum at a particular amount of N-CDots and then is reduced when the N-CDot amount increases further. In contrast, the luminous flux decays exponentially upon increasing the amount of N-CDots from 15 to 50 µL (Figure 3). However, the probed configuration contains only blue light from the LED chip and green light from the N-CDots, whereas white light is actually composed of three main colors, red–green–blue (RGB). Hence, although intended to be a WLED, this configuration has a red deficiency. Therefore, to generate white light at a high luminous flux while keeping the high CRI and LER values and a low CCT, one can integrate red phosphors of high quantum yield into the composite, especially at the low N-CDot amount. Figure 4a presents the PL spectrum of the drop-cast composite films (on the glass slide) containing different amounts of both the N-CDots and the red phosphors in the PVP matrix. The details of the sample composition are given in Table S1, Supporting Information File 1. The amount of red phosphor that was integrated into the system was kept at 5 mg for all samples except for CDR 3 (Figure 4). For CDR 1, CDR 2 and CDR 4, upon variation of the N-CDot amount, we observed a behavior of the PL intensity comparable to that seen in Figure 3. Sample CDR 3 obviously reveals the effect of the red phosphor. In the former case, the total PL intensity of the produced light decreases as the N-CDot amount is increased (at fixed amount of red phosphor). This behavior possibly results from aggregation-induced luminescence quenching and enhanced scattering of the light within the composites. In the case of the sample CDR 3, adding more red phosphor at the fixed N-CDots amount leads to a dramatic increase of the emission of the red phosphor, which is located at 610 nm, and a simultaneous decrease of the blue light emission. At the same time, the green emission of the N-CDots is also reduced, because it may be partially absorbed by the red phosphor whose absorption range covers both the blue and the green range. The optical properties of the generated white light are presented in Figure 4b. The highest CRI of 86 is obtained for the sample CDR 1, while CDR 2 and CDR 3, having lower shares of N-CDots, showed the lowest CRI of 74 and 73, respectively. At the highest amount of N-CDots (CDR 4), the CRI reaches 83. Meanwhile, all samples but CDR 2 provided good CCT values, which are 4900 K, 4000 K and 6000 K for CDR 1, CDR 3 and CDR 4, respectively. These values indicate that almost warm white-light properties are achieved. The highest LER value of 228 lm/W is observed for CDR 1, while CDR 3 and CDR 4 yield 223 lm/W and 212 lm/W, respectively. However, CDR 2 provides the lowest LER of 199 lm/W. Finally, the luminous flux of the white light generated by these samples reaches 63.5 lm for CDR 2, which is the highest, and 61.2 for CDR 3. These samples contain the lowest amounts of N-CDots, such that higher luminous flux values are indeed expected. On the other hand, as the N-CDot amount increases, the luminous flux is reduced. It is reduced to 46 lm for CDR 1 and to 41.9 lm for CDR 4. The CIE color coordinates of these color conversion layers are presented in Figure 4c. Only CDR 2 shows a deviation towards the blue from the complete white region at the center (0.33, 0.34). The remaining samples are very close to the white region, especially CDR 1 and CDR 4, which have coordinates of (0.35, 0.36) and (0.32, 0.34), respectively. Therefore, the light generated using these samples can, in principle, be considered as a good approach to produce white light.

So far, we have investigated the white-light properties of WLEDs comprised of drop-cast PVP/N-CDot composite films with and without red phosphor. In addition to preparing PVP/N-CDot composite films via drop-casting, one can also produce PVP/N-CDot composite fibers via electrospinning. The electrospinning technique is a well-known and facile procedure that leads to the formation of thin fibers by applying a high potential difference between the nozzle of a solution container and the associated grounded conductive collector [61]. Although it showed the worst white-light properties of the WLEDs tested (actually without valid CRI and CCT, Figure 3), here, the PVP/N-CDot mixture containing the lowest amount of N-CDots (15 µL) was employed for the electrospinning. The composition of the PVP/N-CDot mixture used to produce the fibers is identical with the one that was drop-cast to yield the PVP/N-CDot composite films. Figure S4 of Supporting Information File 1 depicts the SEM micrographs of the produced PVP/N-CDot fibers. A detailed description of the characteristics of the fibers is found in the Supporting Information File 1 (Section III).

In general, one can produce 50–100 mg of fibers during a single electrospinning process. Since one color conversion layer contains between 20 and 30 mg of PVP/N-CDot fibers, it is possible to fabricate more than three or four individual color conversion layers using the fibers obtained from one single electrospinning process. In contrast, only a single layer is obtained at a time via the drop-casting approach. Furthermore, instead of directly varying the amount of N-CDots (µL) contained in the color conversion layers, as it was done in the previous measurements, one can adjust the properties of the generated white light by tuning the amount of fibers in terms of their mass (m). More precisely, since the PVP/N-CDot fibers were fabricated using a fixed amount of N-CDots (15 µL), one can indirectly adjust the percentage of N-CDots in the color conversion layers by varying the amount of fibers employed for its production, for instance, the total amount of N-CDots (15 µL) is reached if one uses all of the produced fibers at once.

**Figure 5:**
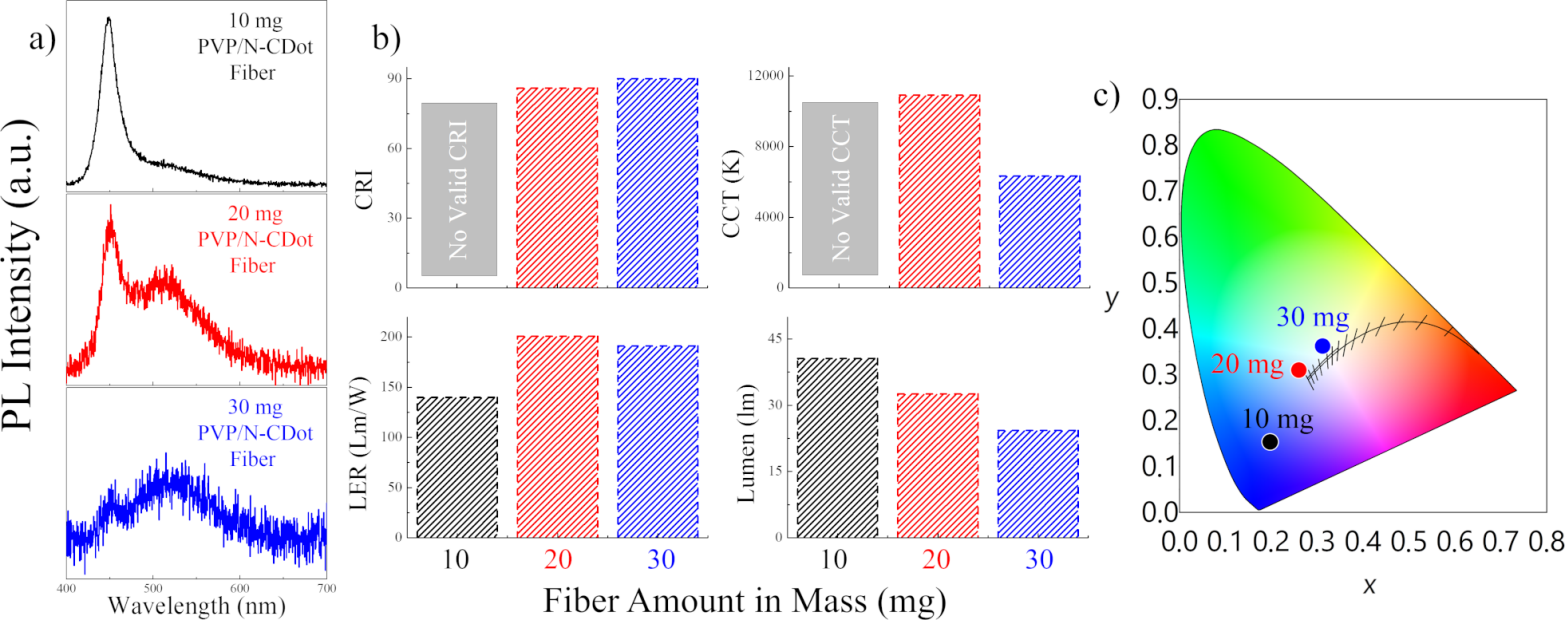
(a) PL spectra of the white light generated by employing color conversion layers with different amounts of PVP/N-CDot fibers coupled to a 450 nm blue LED chip. (b) The calculated CRI, CCT, LER and luminous flux values of the corresponding PVP/N-CDot fibers. (c) The CIE color coordinates of the white light generated by the WLED comprising varying amounts of the PVP/N-CDot fibers.

In this sense, three different color conversion layers varying in their fiber mass have been prepared. Then, each sample, corresponding to a particular fiber mass, was squeezed between two thin glass slides. The corresponding spectra are presented in Figure 5 together with the resulting optical properties including CRI, CCT, LER and luminous flux. The PL spectra of the white light generated by employing color conversion layers with different amounts of PVP/N-CDot fibers (Figure 5a) indicate that the ratio of the blue and green emission bands is dramatically reduced as the fiber amount increases, comparable to the results derived from Figure 3. The PL spectrum of the WLED containing 10 mg of the fibers does not show any particular green emission band and is rather dominated by the blue emission coming from the blue LED chip. In the case of the color conversion layers comprising 20 mg of the fibers, the green emission of the N-CDots reaches almost the same intensity as the blue emission, and the green emission becomes more intense than the blue when the fiber amount is set to 30 mg. Hence, without dealing with the N-CDot amount directly, one can tune the spectral shape of the generated white light just by adjusting the mass of the fibers integrated into the WLEDs. The resulting optical properties are given in Figure 5b. The WLEDs containing 10 mg of fiber yield no valid CRI and CCT values since the ratio of the blue and green emission is inadequate for producing satisfactory white light. On the other hand, as the fiber content increases, the CRI becomes 86 at 20 mg and reaches a value of 90 at 30 mg, indicating very good white-light properties suitable for possible white-light applications. Meanwhile, the CCT value is 11000 K at 20 mg and reduces dramatically to 6000 K as the fiber amount is increased to 30 mg. Thus, one can adjust the generated white light from a cool white to a warm white simply by incorporating more fibers into the color conversion layers. Again, we would like to stress that these PVP/N-CDot composite fibers were prepared using the N-CDot mixture that yielded no valid CRI and CCT when processed into a drop-cast film – yet, high CRI and low CCT values could be achieved here by adjusting the amount of the fibers incorporated into the WLEDs. The highest CRI and the lowest CCT values are obtained from the WLEDs with 30 mg of the fibers and are comparable to the values obtained using the drop-cast films containing the highest amount of PVP/N-CDots (50 μL, Figure 3). In summary, using electrospun fibers allows one to reduce the amount of N-CDots used and to obtain adequate white-light properties. These results can be explained by the significantly enhanced multiple-scattering in the conversion layers containing the composite fibers due to their nonuniform structure full of scattering centers. As a consequence, more N-CDots can interact with the incoming blue light thanks to the multiple-scattering in the layers, and the N-CDot emission is reinforced. Moreover, the LER of the generated white light improves upon adding more fibers to the color conversion layers. For instance, the LER is 140 lm/W at 10 mg and reaches up to 201 lm/W at 20 mg. However, in line with the results derived from Figure 3, the LER decreases again when more fibers are included in the color conversion layers. This is due to the inadequate *S*/*N* level as the PL intensity is dramatically reduced. The PL intensity loss can be retraced in terms of the luminous flux, which is also a function of the fiber amount. Going from 10 mg to 30 mg of incorporated fibers, the total visible light output of the WLEDs reduces from 41 to 24 lm, corresponding to a reduction of almost 41%. This effect supports the proposed idea that incorporating a larger amount of fibers causes an enhancement of the scattering of the blue light within the color conversion layers and thus diminishes the PL intensity. The CIE color coordinates presented in Figure 5c proceed from blue to white (center values) when increasing the fiber amount from 10 mg to 30 mg. More precisely, the WLEDs containing 10 mg of fiber have a CIE of (0.20, 0.15), while the CIE is shifted towards the white at 20 mg (0.26, 0.31) and almost reaches the center at 30 mg (0.31, 0.36), indicating that white-light properties are gradually achieved.

## Conclusion

In this study, various forms of N-CDots have been employed as water-based color-conversion layers over blue LED chips to generate white light. We observed that drop-cast PVP/N-CDot composite films show promising white-light properties at higher N-CDot concentrations. In addition, since the white light generated using these films suffers from a red deficiency, commercial red phosphor can be integrated into the PVP/N-CDot composite films. The additional red emission improves the LER and the luminous flux, and as a consequence, satisfying white-light properties can be achieved at a lower amount of N-CDots. Additionally, PVP/N-CDot composite fibers were produced and successfully employed as color conversion layers over a blue LED chip. We produced electrospun composite fibers that contain only a small amount of N-CDots (in total 15 μL), and we were able to adjust the properties of the generated white light by varying the amount of the fiber within the color conversion layers, finally achieving a CRI of 90 and a CCT of 6000 K. This result is surprising because the fibers were produced using a N-CDot solution (with a low percentage of N-CDots) which yielded no valid CRI and CCT values when processed into a drop-cast composite film. Hence, using fibers, one can significantly reduce the amount of N-CDots used. In summary, the N-CDot-based generation of white light with satisfactory white-light properties was explored by employing different forms of color conversion layers. The white light resulting from the probed configurations showed satisfactory characteristics in terms of CRI, CCT, LER and luminous flux, which makes N-CDots a promising material for use in phosphor-converted WLED applications.

## Supporting Information

Section I – XPS full scan spectrum of nitrogen-doped CDots; high-resolution C 1s, N 1s, and O 1s XPS spectra of nitrogen-doped CDots; FTIR, absorption and PL spectra of N-CDots in solution; Section II – Response of the varying amount of N-CDots in terms of CRI, CCT, LER, and luminous flux as a function of driving current; Section III – SEM images of N-CDot fibers with a magnification of 10000× and 25000×; Section IV – A table with the amounts of added N-CDots and red phosphor for each sample.

File 1 Additional experimental data.
