# Supplementary material for: Optimization and performance of nitrogen-doped carbon dots as a color conversion layer for white-LED applications
Source: Beilstein J Nanotechnol. 2019 Oct 15;10:2004–13. doi: 10.3762/bjnano.10.197 (PMC6808204; doi:10.3762/bjnano.10.197)
Supplement: File 1 — Additional experimental data. [file Beilstein_J_Nanotechnol-10-2004-s001.pdf]

BEILSTEIN JOURNAL OF NANOTECHNOLOGY

Supporting Information

for

Optimization and performance of nitrogen-doped carbon dots as a color conversion layer for white-LED applications

Tugrul Guner, Hurriyet Yuce, Didem Tascioglu, Eren Simsek, Umut Savaci, Aziz Genc, Servet Turan and Mustafa M. Demir

Beilstein J. Nanotechnol. **2019,** *10,* 2004–2013. doi:10.3762/bjnano.10.197

Additional experimental data

License and Terms: This is a supporting information file under the terms of the Creative Commons Attribution License (http://creativecommons.org/licenses/by/4.0). Please note that the reuse, redistribution and reproduction in particular requires that the authors and source are credited.
The license is subject to the *Beilstein Journal of Nanotechnology* terms and conditions: (https://www.beilstein-journals.org/bjnano)

SECTION I

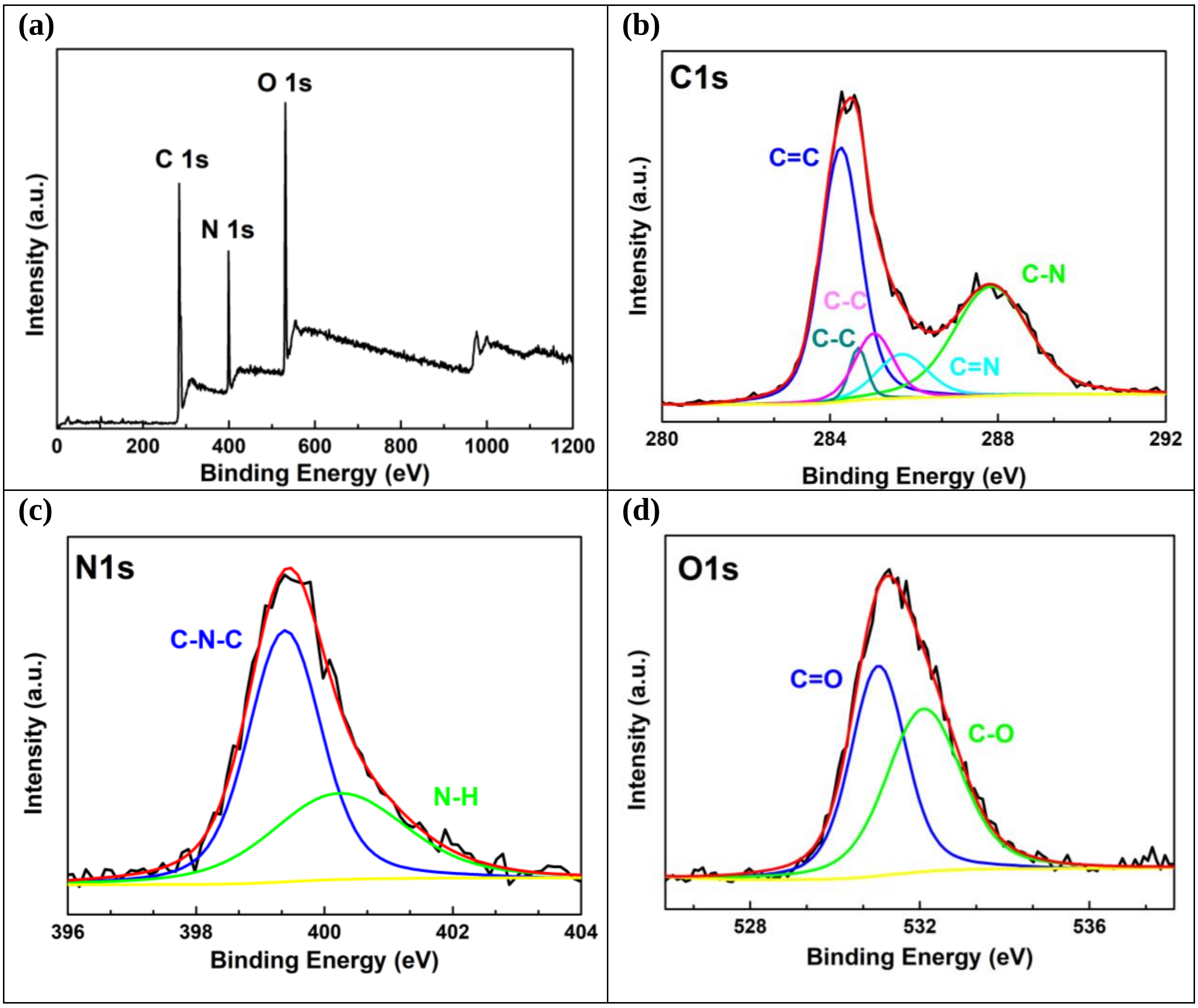

Figure S1: (a) XPS full scan spectrum of nitrogen doped CDots. High resolution (b) C 1s (c) N 1s (d) O 1s XPS spectra of nitrogen doped CDots.

X-ray photoelectron spectroscopy (XPS) was used to investigate the composition and surface groups of the CDots. The wide XPS spectrum of the CDots as shown in Figure S1a shows that carbon (284.39 eV), nitrogen (399.28 eV), and oxygen (531.23 eV) are presented at the surface of CDots. The C 1s spectrum (Figure S1b) has five components, which can be deconvoluted into several peaks corresponding to C=C (sp^2 carbon) (284.26 eV), C–C (sp^3 carbon) (284.67 eV, 285.05 eV), C=N (285.72 eV), and C–N (287.84 eV). On the other hand, N 1s spectrum (Figure S1c) can be deconvoluted into two components having peaks at 399.39 and

400.23 eV due to the pyridinic N (C–N–C) and pyrrolic N (N–H) groups, respectively. There are also oxygen containing groups, which are detected at the surface of CDots (Figure S1d). Corresponding O 1s band contains two signals that can be associated with sp^2 (C=O, 531.02 eV) and sp^3 (C–O, 532.09 eV) [1,2]. XPS data presented here hints about the presence of nitrogens.

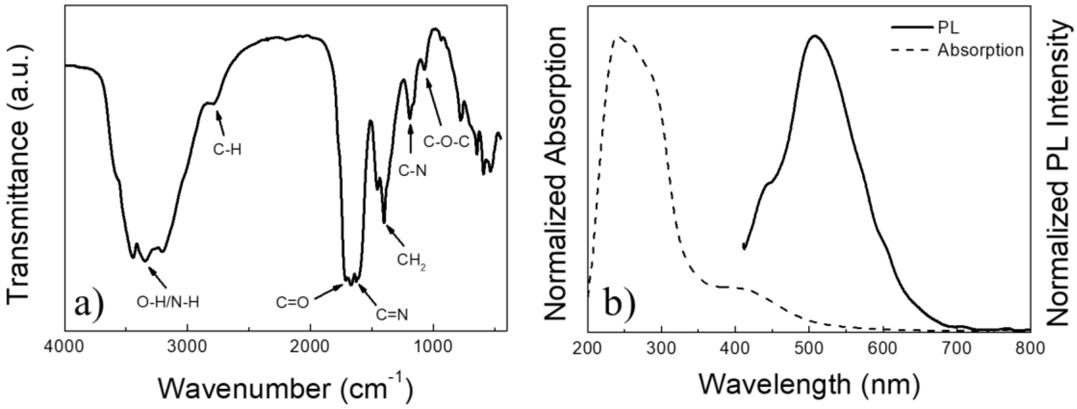

Figure S2: (a) FTIR, (b) Absorption (dashed line; left vertical axis) and PL (solid line; right vertical axis) spectra of N-CDots in solution.

In order to confirm whether the material is nitrogen-doped or not, it was further characterized by FTIR spectrum (Figure S2a). The broad band which is centred at 3346 cm^{-1} belongs to the O–H and symmetric/asymmetric N–H bonds [3]. There is a weak signal at 2787 cm^{-1} exhibiting the presence of C–H stretching vibration. Moreover, FTIR spectrum indicates that there are amide bonds, where their presence can be inferred from their characteristic vibrational signals: 1700 and 1639 cm^{-1}, which correspond to C=O and C=N stretching vibrations, respectively. The other amide band, which is C–N stretching, is observed at 1192 cm^{-1}. In addition, the characteristic peaks at 1400 and 1069 cm^{-1} belong to the CH_2 bending and C–O–C stretching vibration [3,4]. As a result, based on the both XPS and FTIR data, the material that have been

employed throughout this study can be confirmed as it is a nitrogen-doped carbon dots (N-CDots).

Photoluminescence (PL) and absorption spectrum of N-CDots were investigated and presented in Figure S2b. There are two strong absorption peaks: π–π* transition of aromatic sp^2 domains at 234 nm and the electronic transition of C-C in the excited N-CDots at 260 nm [5,6]. Another one is a broad peak at 405 nm, which corresponds to n–π transition of the nitrogen-doped CDots by the surface states [6]. In this study, 450 nm LED chip was employed as excitation source, and in return, the strong emission of N-CDots was appeared around 506 nm lying in the green region. This emission range of the N-CDots makes it possible to be used as color conversion layer over blue LED chip in phosphor-converted white LED type configurations.

References

[1] Khan, W.U., et al. High Quantum Yield Green-Emitting Carbon Dots for Fe (III) Detection, Biocompatible Fluorescent Ink and Cellular Imaging. Scientific reports 2017; 7(1): 14866.

[2] Siddique, A.B., A.K. Pramanick, S. Chatterjee, and M. Ray. Amorphous Carbon Dots and their Remarkable Ability to Detect 2, 4, 6-Trinitrophenol. Scientific reports 2018; 8(1): 9770.

[3] Feng, Z., Z. Li, X. Zhang, Y. Shi, and N. Zhou. Nitrogen-Doped Carbon Quantum Dots as Fluorescent Probes for Sensitive and Selective Detection of Nitrite. Molecules 2017; 22(12): 2061.

[4] Niu, J., et al. Facile synthesis and optical properties of nitrogen-doped carbon dots. New Journal of Chemistry 2014; 38(4): 1522-1527.

[5] Lin, H., L. Ding, B. Zhang, and J. Huang. Detection of nitrite based on fluorescent carbon dots by the hydrothermal method with folic acid. Royal Society open science 2018; 5(5): 172149.

[6] Hou, J., et al. Synthesis and formation mechanistic investigation of nitrogen-doped carbon dots with high quantum yields and yellowish-green fluorescence. Nanoscale 2016; 8(21): 11185-11193.

SECTION II

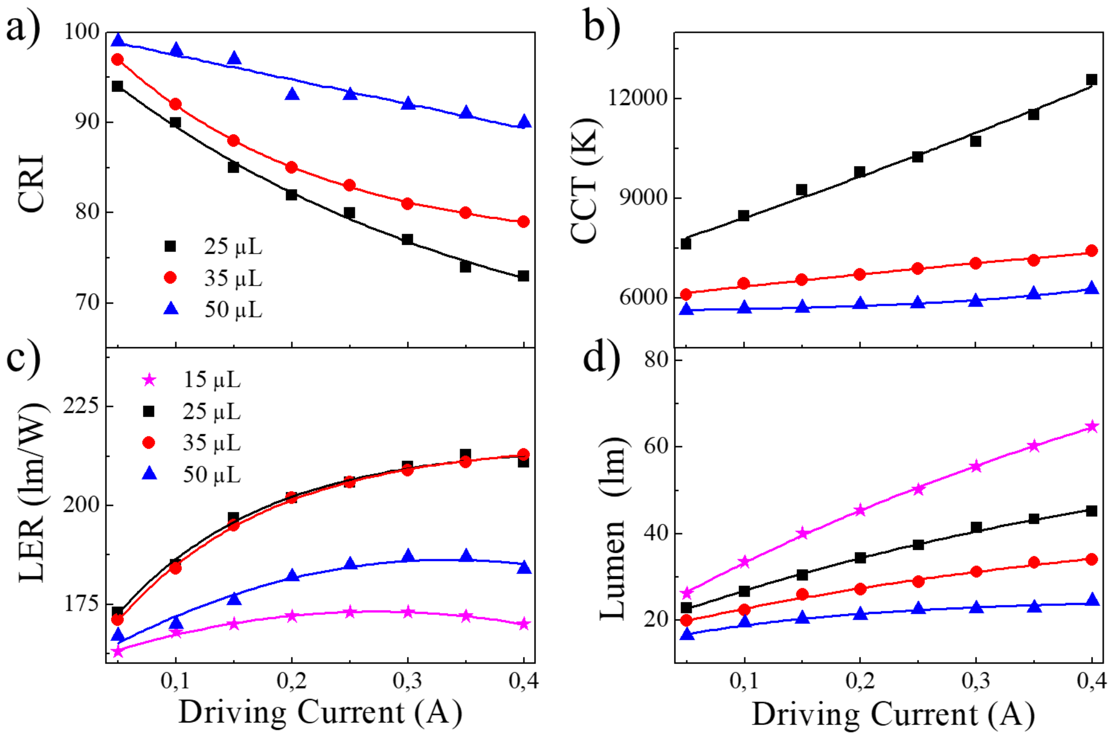

Figure S3: Response of the varying amount of N-CDots in terms of (a) CRI, (b) CCT, (c) LER, and (d) Lumen as a function of driving current.

Since there is no any valid data in terms of CRI and CCT for the N-CDot amount of 15 µL, first two figures, Figure S3a and S3b, contain the response of N-CDots having 25, 35, and 50 µL amounts against the driving current. Figure S3a indicates that there are three different decaying curves for CRI. Among those, composite containing 25 µL N-CDots, presented with black squares shows the highest reduction from 94 to 73. Meanwhile, composite containing 35 µL (red circles) decays from 97 to 79, 50 µL (blue triangles) decays from 99 to 90 showing the least reduction for CRI compared to other amounts. On the other hand, there is a strict increase of CCT for composite containing 25 µL N-CDots from 7600 K to 13000 K as the driving current increases while the other amounts follow just a slightly increasing pattern indicating a good stability. So far, there is no any valid CRI, and CCT were obtained for the composite containing

15 µL N-CDots. On the contrary, meaningful results were registered in the case of lumen and LER at this amount of N-CDots, which were marked with pink stars. It shows the lowest level of variation in LER (Figure S3c), and highest in lumen (Figure S3d) compared to other samples. It is expected since PVP/N-CDot (15 µL) composite has a small number of green-emitting N-CDots that distributed through the composite, which also leads to a low level of scattering of blue light. Meanwhile, 25 and 35 µL containing composites provided the highest LER (Figure S3c), which increase from 175 to 212 lm/W as the driving current increases. By increasing the N-CDot amount more (at 50 µL), LER drops dramatically, and it shows variation against driving current below the level of 190 lm/W. All N-CDot amounts follow a saturating pattern indicating driving current can effectively improve the LER until to some point, which is 0.30–0.35 A for these samples. In the case of lumen, as presented in Figure S3d, all composites follow a strictly increasing curve as the driving current increases, which is due to rising LED emission intensity at the same time. It reaches to its highest value at 15 µL while to lowest at 50 µL. Not surprisingly, higher amount means higher absorption/emission and scattering lead to reduction in total output PL intensity of the produced white light.

SECTION III

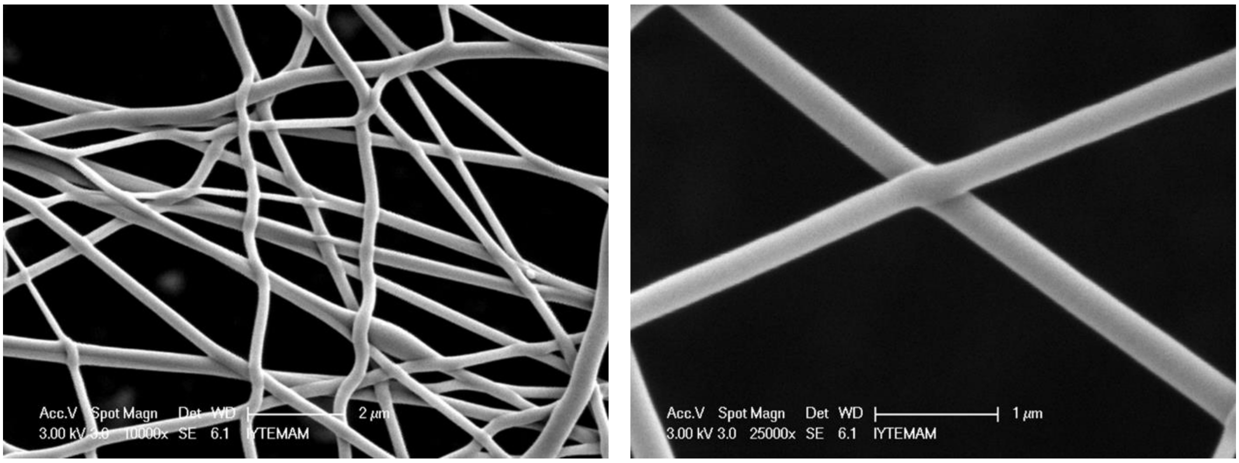

Figure S4: SEM images of N-CDot fibers with the magnification of (a) 10000x, and (b) 25000x.

First micrograph (left) hints about the overview of the obtained fiber distribution. Morphologically, fiber formation is achieved since no any bead formation is presented in the image. Apart from their morphology, the fibers show branched distribution along the image revealing the formation of fiber network. Second, micrograph focuses on individual fibers capturing at higher magnification. In this micrograph, the fibers appear as they have been formed smoothly with diameters of ≈300 nm.

SECTION IV

Table S1: The table shows the amounts of added N-CDots and red phosphor for each sample.

Sample	N-CDot (µL)	Red Phosphor (mg)
**CDR 1**	**25**	**5**
**CDR 2**	**15**	**5**
**CDR 3**	**15**	**10**
**CDR 4**	**35**	**5**
